# CD38: An Immunomodulatory Molecule in Inflammation and Autoimmunity

**DOI:** 10.3389/fimmu.2020.597959

**Published:** 2020-11-30

**Authors:** Zayda L. Piedra-Quintero, Zachary Wilson, Porfirio Nava, Mireia Guerau-de-Arellano

**Affiliations:** ^1^ School of Health and Rehabilitation Sciences, Division of Medical Laboratory Science, College of Medicine, Wexner Medical Center, The Ohio State University, Columbus, OH, United States; ^2^ Biomedical Science Undergraduate Program, College of Medicine, Wexner Medical Center, The Ohio State University, Columbus, OH, United States; ^3^ Departamento de Fisiología, Biofísica y Neurociencias, Centro de Investigación y de Estudios Avanzados (CINVESTAV), México City, México; ^4^ Institute for Behavioral Medicine Research, The Ohio State University, Columbus, OH, United States; ^5^ Department of Microbial Infection and Immunity, The Ohio State University, Columbus, OH, United States; ^6^ Department of Neuroscience, The Ohio State University, Columbus, OH, United States

**Keywords:** CD38, ADP-ribosyl cyclase, cADPR hydrolase, inflammation, autoimmunity, NADase

## Abstract

CD38 is a molecule that can act as an enzyme, with NAD-depleting and intracellular signaling activity, or as a receptor with adhesive functions. CD38 can be found expressed either on the cell surface, where it may face the extracellular milieu or the cytosol, or in intracellular compartments, such as endoplasmic reticulum, nuclear membrane, and mitochondria. The main expression of CD38 is observed in hematopoietic cells, with some cell-type specific differences between mouse and human. The role of CD38 in immune cells ranges from modulating cell differentiation to effector functions during inflammation, where CD38 may regulate cell recruitment, cytokine release, and NAD availability. In line with a role in inflammation, CD38 appears to also play a critical role in inflammatory processes during autoimmunity, although whether CD38 has pathogenic or regulatory effects varies depending on the disease, immune cell, or animal model analyzed. Given the complexity of the physiology of CD38 it has been difficult to completely understand the biology of this molecule during autoimmune inflammation. In this review, we analyze current knowledge and controversies regarding the role of CD38 during inflammation and autoimmunity and novel molecular tools that may clarify current gaps in the field.

## Introduction

For the last four decades, the contribution of CD38 to cell biology and CD38’s links to human disease have been the focus of substantial research. CD38 was initially described as a surface protein in T cells capable of inducing cell activation (1, 2). Soon after, CD38 expression was also reported in other immune cells, such as B cells, natural killer (NK) cells, neutrophils, and myeloid cells (1, 3–6). Likewise, roles for CD38 in cell differentiation, cytokine release, migration, and apoptosis processes were revealed (7–11). At the molecular level, the similarity of CD38 with a soluble enzyme purified from the mollusk *Aplysia californica* led to the characterization of CD38 as an ADP-ribosyl cyclase and a cyclic ADPR (cADPR) hydrolase that utilizes NAD as substrate (12, 13).

Since these findings were reported, many studies have sought to understand the extent to which CD38 contributes to the development of inflammatory and autoimmune disease, *via* modulation of immune responses. So far, it is known that CD38 expression is robustly induced in immune cells after activation and regulates infection-induced inflammatory processes, from cell recruitment to induction of adaptative immune responses (5, 14). Nonetheless, the mechanisms utilized by CD38 to mediate each stage of inflammation are still poorly understood. The function of CD38 during inflammatory autoimmunity has also been the subject of many studies in several diseases. Depending on the disease type, immune cell population, or animal model analyzed, several reports indicate that CD38 can either suppress or induce autoimmunity. The lack of consensus highlights the need of more research in order to understand the biology of CD38 and its contributions to inflammation and autoimmunity.

In the following sections, we summarize the studies available on the role of CD38 during inflammation and autoimmune disease. In addition, we present a comprehensive summary of molecular tools available for the study of CD38 that should help advance our understanding of CD38’s role in physiology and disease.

### CD38: Function, Structure, and Localization

CD38 is a protein of 300 amino acids encoded by homologous genes located on chromosome 4 and 5 in humans and mice, respectively (15). Within the cell, CD38 is often found localized on the cell surface, but it can also be detected in intracellular compartments such as the endoplasmic reticulum, nuclear membrane and mitochondria (16–19). Structurally, CD38 is a single chain glycoprotein with a single transmembrane segment and can topologically behave as a type II or type III membrane protein depending on its membrane orientation. In the most common type II orientation, CD38’s short amino tail faces into the cytosol while CD38’s catalytic domain faces the extracellular environment (20, 21). A type III orientation, with the catalytic domain facing the cytosol, has been also reported (17, 22). These two orientations have functional implications, given that CD38’s enzymatic substrates and products would be consumed and produced in the extracellular or the intracellular compartments. CD38 catalyzes the synthesis of nicotinamide (NAM) and ADPR using nicotinamide adenine dinucleotide (NAD^+^) as a substrate. NAD^+^, an essential cofactor that regulates energy metabolism (23), can be converted to cADPR with the release of NAM. Interestingly, cADPR can also be hydrolyzed to ADP-ribose by CD38. Thus, CD38 has both ADP-ribosyl cyclase and cADPR hydrolase enzymatic activities. Both ADPR and cADPR act as second messengers controlling several cell functions through calcium (Ca^2+^) mobilization (24, 25). Therefore, the implications of these observations in cell physiology have received significant interest. Besides its enzymatic function, CD38 can also act as a receptor to CD31 (26). Through the latter interaction, CD38 could act as an adhesion molecule mediating selectin-like binding of hematopoietic cells to endothelial cells and facilitating their transmigration to tissue (27, 28).

### CD38 Distribution in the Immune System

**Figure 1:**
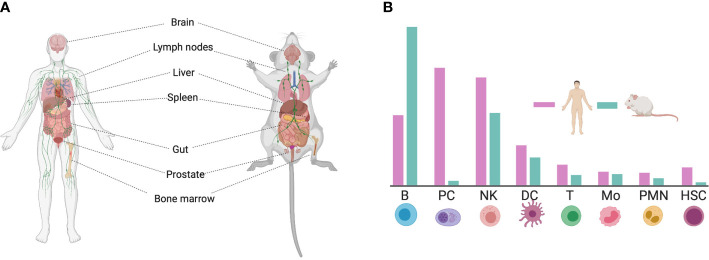
Expression of CD38 in human and mouse tissue. **(A)** CD38 is ubiquitously expressed in the human and mouse body. **(B)** Schematic representation of CD38 expression on immune cells. The differences in CD38 expression between human and mouse is highlighted [adapted from (29, 30)]. This figure was created using Biorender (https://biorender.com). B, B cell; PC, Plasma cell; NK, Natural Killer cell; DC, Dendritic Cell; T, T cell; Mo, Monocyte; PMN, Molymorphonuclear cell; HSC, Hematopoietic Stem Cell.

CD38 is a ubiquitous protein expressed in multiple tissues. Non-hematopoietic tissue expression include prostatic epithelial cells, pancreatic islet astrocytes, smooth muscle cells, retinal tubes, kidney, gut, and brain in both mice and humans (**Figure 1A**) (31–34). However, CD38 is most highly expressed in hematopoietic tissues such as the bone barrow and lymph nodes (35). Within immune cells, CD38 is highly expressed in B cells, macrophages, dendritic cells (DCs), innate lymphoid cells (ILC), natural killer (NK) cells, T cells, neutrophils, and monocytes (36). Nevertheless, the level of CD38 expression among these populations may differ between human and mouse, as observed in a transcriptional comparison between species (29, 30) (**Figure 1B**).

Based on the pattern of CD38 expression during these cells’ life cycle, CD38 may impact a range of processes, from differentiation to effector function. CD38 is expressed in B cell precursors, germinal center B cells, and plasma cells (37, 38). However, some differences in expression have been reported between human and mouse (29, 39). For instance, human progenitor B cells express CD38 on surface but lose this expression after maturation whereas murine B cells express CD38 throughout its entire differentiation process. Also, CD38 is highly expressed on B cells from germinal center while expression on murine B cells is low. In contrast, only human plasma cells have been reported to express CD38. Something similar is observed in early human T cells precursors and double-positive CD4^+^CD8^+^ thymocytes (DP), which display high expression of CD38, while mouse DP and resting T cells do not express CD38 (8, 29, 38, 40–43). In contrast, both mature human and mouse T and B cells induce CD38 upon activation. Therefore, this molecule is often used as a cell activation and differentiation marker (7, 8, 14). Likewise, expression of CD38 in NK cells has been linked to activation, induction of cytotoxic activity, and secretion of IFN-*γ*. It has been demonstrated that CD38 controls these cellular processes through its receptorial activity, a function dependent of CD38’s lateral association with CD16 (3, 44). In the myeloid linage, CD38 expression is induced after activation in inflammatory conditions in both mice and humans. CD38 appears to regulate cytokine release, adhesion, and cellular migration toward sites of inflammation (4, 5, 33, 45, 46). Thus, the prominent expression of CD38 in immune cells suggests important roles in immune responses, ranging from the development of inflammation in response to infection to development or regulation of adaptive immune responses.

## Inflammation

Inflammation is a characteristic body response to harmful stimuli aimed at eliminating the source of damage and returning the tissue to functional homeostasis (47). Eliciting stimuli include pathogen-associated molecular patterns (PAMPs) from infectious pathogens and damage-associated molecular patterns (DAMPs) released during sterile tissue/cell injury during traumatic injuries or autoimmune disease (48–52).

Recognition of PAMP/DAMPs by pattern recognition receptors (PRRs), present in innate immune cells, induces the synthesis of lipid inflammatory mediators such as arachidonic acid (AA). AA is then converted to prostaglandins (PG) and leukotrienes (LT) that act as potent neutrophil chemoattractants (53, 54) and trigger inflammation. The inflammatory process is a multistep event encompassing 1) endothelial adhesion, 2) endothelial cell transmigration, 3) chemotaxis, 4) cytokine/chemokine release to recruit additional immune cells (such as monocytes), 5) phagocytosis, and 6) antigen presentation leading to initiation of antigen-specific adaptive immune responses (55, 56). The first wave of cells reaching the tissue is formed by neutrophils, which are recruited in response to PG and LT with robust neutrophil chemoattractant activity. When neutrophils reach the infected/injured tissue, they release inflammatory mediators that recruit a secondary wave of monocytes. Monocytes can differentiate into macrophages (MØ) and dendritic cells. Monocyte derived-macrophages produce additional inflammatory cytokines and, together with neutrophils and DCs, phagocytose pathogens or dying cells (57). After phagocytosis, professional antigen presenting cells (APCs) such as macrophages and DCs process antigens and load them onto Major Histocompatibility Complex (MHC) molecules. These antigen-loaded MHC molecules are presented on the cell surface for recognition by antigen-specific T cells. Thus, this step marks the initiation of antigen-specific adaptive immune responses (58). The combined effects of immune cells and their mediators generally resolve inflammation and initiate tissue repair. However, if the inflammatory process is not resolved, it can lead to chronic inflammation. Lack of inflammation resolution may be due to the continuous presence of self-antigens, as in autoimmune diseases (59).

CD38 is robustly induced during infection and the ensuing inflammation (5, 45, 60). The human CD38 gene promoter includes putative binding sites for the transcription factors Sp1, Retinoic Acid Responsive Elements (RARE) and IRF-1 (61). Thus, has been reported that CD38 expression is under the transcriptional control of IFN type I and II as well as TNF-α/NF-κB stimulation (61–63). Therefore, CD38 has been clearly linked to inflammation and has been the subject of considerable study, particularly in the context of infection. In the sections below, we will summarize our current knowledge on the contribution of CD38 to the various steps of the inflammatory process.

### CD38 Regulates Cellular Migration

**Figure 2:**
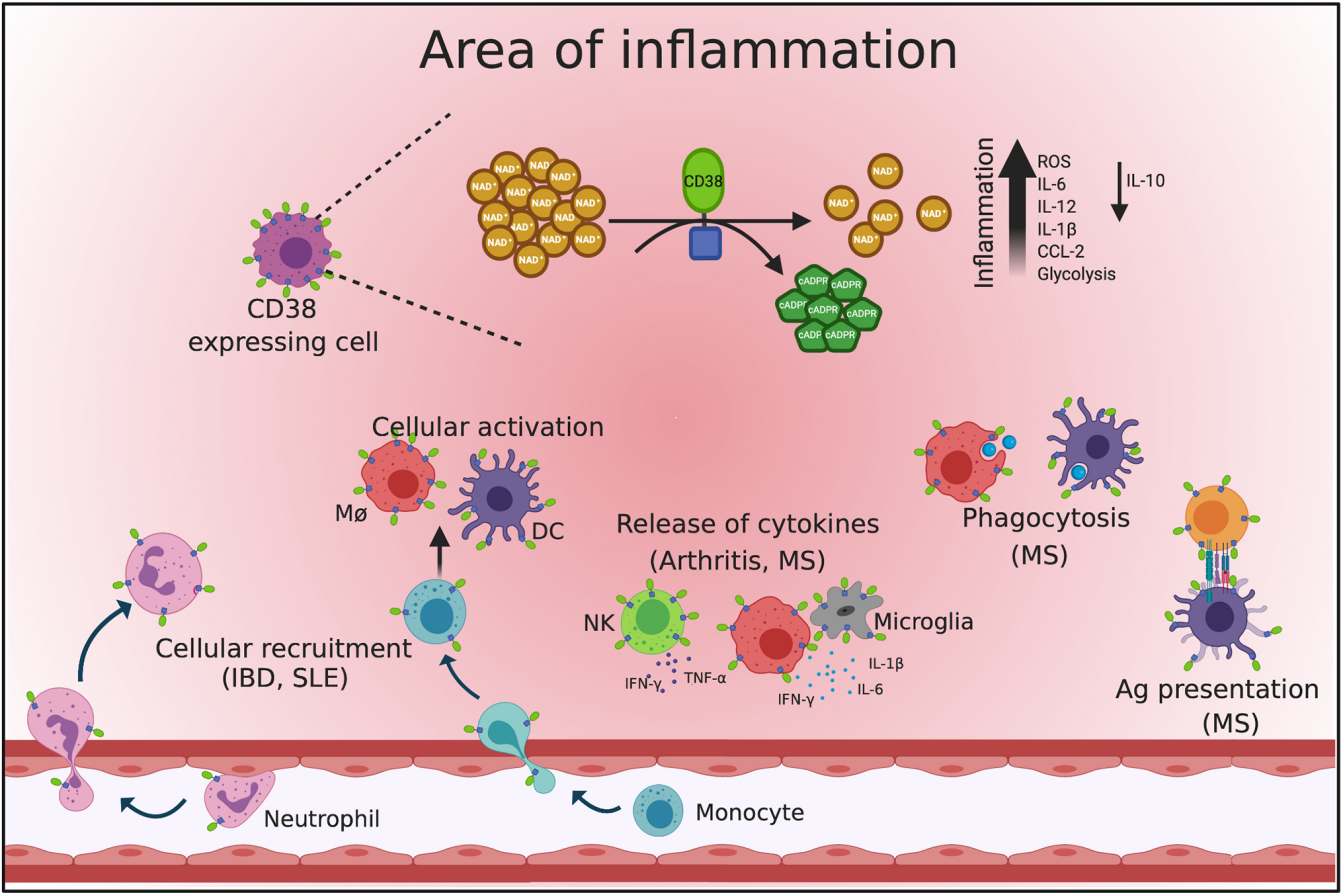
Role of CD38 during inflammation. The figure displays a schematic representation of an inflammatory process and the events impacted by CD38. During inflammation, CD38 can modulate cell recruitment, cytokines and chemokines release, cell activation, phagocytosis, and antigen presentation. CD38 expressing cells consume NAD^+^ to produce cADPR an event that leads to inflammation. This figure was created using Biorender (https://biorender.com).

The role of CD38 during infection-induced inflammation has been the focus of substantial study, revealing a supporting role for CD38 during inflammation (**Figure 2**). Transmigration through endothelial cells and chemotaxis toward the site of injury is one of the first steps in the inflammatory process (64, 65). CD38 appears to be essential for both of these processes, as deletion of CD38 in mouse impairs the recruitment of immune cell from blood to sites of infection or tissue injury (33, 45, 66). Furthermore, induction of CD38 expression in hematopoietic cells such as neutrophils, monocytes, dendritic cells, and macrophages is observed in response infection or cellular activation (5, 9, 45, 60).

The effects of CD38 on transmigration may be mediated by the interaction of CD38 with its cognate receptor CD31 (27). CD31 constitutes the major endothelial adhesion molecule involved in transendothelial migration (67). Thus, immune cells expressing CD38 may adhere to endothelium and mediate transmigration through CD31. This has been demonstrated for neutrophils in the context of infection. *Listeria monocytogenes* and *Streptococcus pneumoniae* infections normally result in neutrophil recruitment, but this recruitment is impaired in CD38 deficient mice (45, 68). CD38 also appears to mediate the migration of other immune cells. For instance, CD38 is essential for T cell and macrophage infiltration into the brain in a mouse model of cerebral ischemia (9).

After transmigration into the tissue, chemotaxis will direct infiltrating cells to the site of injury or infection in response to chemoattractant gradients (69). CD38 also appears to contribute to chemotaxis in both mice and humans, given that neutrophils with low levels of CD38 show defects in chemotactic migration to the chemoattractant N-formyl-methionine-leucyl-phenylalanine (fMLF) (6, 68). Moreover, CD38’s calcium mobilization activity has been shown to control chemokine receptor signaling in neutrophils and dendritic cells. CD38’s enzymatic activity produces cADPR/ADPR, thereby triggering Ca^2+^ release from intracellular stores and Ca^2+^ influx from the extracellular space. Intracellular Ca^2+^ signaling induces expression of chemokine receptors such as CXCR4, CCR7 and N-formyl peptide receptor 1 (FPR1), as well as activation of cytoskeletal and adhesion molecules. Thus, cADPR/ADPR levels regulate cellular chemotaxis in a CD38 dependent manner (68, 70, 71). Also, supporting a role for CD38 in chemotaxis, human chronic lymphocytic leukemia (CLL) cells expressing high levels of CD38 exhibit enhanced *in vitro* migration in response to chemokines. Increased chemotaxis was accompanied by increased intracellular Ca^2+^ and actin polymerization. These events induced cellular spreading and cell migration *via* GTPases Rap1/RasGRP2 activation (72).

Overall, multiple lines of evidence support a role for CD38 during transmigration and chemotaxis of neutrophils and monocytes that can next perform their signature phagocytic function.

### Phagocytosis and Antigen Presentation

Phagocytosis is the process by which immune cells such as neutrophils, macrophages and dendritic cells, engulf and eliminate invading pathogens, foreign particles, and infected or apoptotic cells (73). Phagocytosis requires calcium signaling for cytoskeleton activation and subsequent phagosome maturation (74, 75). Therefore, one obvious way in which CD38 could contribute to phagocytosis is *via* cADPR and NAADP-induced Ca^2+^ signaling (25, 76, 77). Indeed, CD38 is recruited and internalized to the phagocytic cup during macrophage Fc*γ*R-mediated phagocytosis. This process is accompanied by increased extracellular cADPR and intracellular Ca^2+^. More conclusively demonstrating a role, the absence of CD38 in murine macrophages impairs Ca^2+^ signaling and phagocytosis of *Mycobacterium bovis* BCG *in vivo* (76). Similarly, the role of CD38 in controlling *Listeria monocytogenes* infection appears to stem from its role in phagocytic function. The inability of CD38 deficient macrophages to control *Listeria* infection in macrophages is due to impaired phagocytosis, as CD38 deficient-murine macrophages maintain their ability to kill *L. monocytogenes* (45). Thus, CD38 appears to play a key role in phagocytosis at the level of bacterial uptake.

After phagocytosis, internalized protein-antigens are digested into peptide fragments that can be loaded onto MHC molecules (78). Then, the peptide-loaded MHC molecules are transported and displayed on the APC cell surface for T cell recognition and initiation of antigen-specific immune responses (79). The specialized APC-T cell interface formed during antigen presentation is known as the immunological synapse (IS) and involves recruitment and signaling of T cell receptor (TCR), costimulatory and adhesion molecules (80). Remarkably, CD38 has been important for antigen presentation of bacteria in both humans and mice (16, 81) and several reports indicate that CD38 may be regulating the signaling induced at the IS. In support of this idea, the contribution of CD38 to TCR/CD3 complex signaling has been widely demonstrated (2, 82). Further analysis has also revealed that CD38 accumulates through the periphery of the mature T/APC IS, suggesting that this molecule also regulates TCR signaling induced during antigen presentation in a human T cell line (16). Interestingly, surface expression of CD38 in antigen-stimulated T cells correlates with increasing calcium release, consistent with a role of CD38 in antigen induced effector function. Supporting this idea, CD38 blockade with the monoclonal antibody IB6 suppresses antigen-induced IL-12 and IFN-*γ* production, an effect likely mediated by cADPR (16). As mentioned earlier, adhesion and costimulatory molecules expressed by T cells and/or APCs also intervene for an effective IS. The association of CD38 with adhesion and costimulatory molecules suggests that it could also modulate antigen presentation at this level. For example, in APCs, trafficking of MHC-loaded peptides to the cell surface depends of the tetraspanin CD9 (83). Interestingly, CD38 associates with both MHCII and CD9 in membrane rafts of human monocytes from where it induces intracellular Ca^2+^ fluxes (81). Also, monocytes loaded with *Staphylococcus* enterotoxin E require CD38/MHCII/CD9-complex to present the antigen to T cells (81). Similarly, Zilber et al. reported that monocyte surface CD38 acts as an MHCII coreceptor to induce tyrosine phosphorylation of intracellular proteins during superantigen-induced activation (84). In this regard, CD38 has been also reported to associate with CD81 and CCR7, crucial molecules for clustering and signaling activation during T/APC synapses, in human DCs (11, 85, 86). On the other hand, the IS also requires prolonged cell-cell interaction which is dependent of adhesion molecules such as CD11b (87). Co-localization of CD38 and CD11b was also observed in dendritic cells (11). Thus, these observations suggest another important role for CD38 during antigen presentation.

Taken together, these findings indicate that CD38 contributes to phagocytosis and antigen presentation. Through these processes, CD38 may promote antigen-specific adaptive immune responses.

### Cytokine Release

During inflammation, immune cells release inflammatory mediators to eliminate pathogens (88), a process modulated by CD38 expression. For instance, CD38 has been shown to limit bacterial infection, as evidenced by increases susceptibility to *Salmonella typhimurium* infection in CD38-deficient macrophages. Furthermore, the inflammatory response against *S. typhimurium* was also impacted, with notable reductions in pro-inflammatory cytokines IL-1β, IL-6, IL-12, and TNF-α (89). Similar results have been observed in human primary macrophages, where impaired secretion of IL-12, and IL-6 was observed after CD38 chemical or genetic inhibition (5). The implication of CD38 in cytokine release during viral infections was been also shown (62). During Respiratory Syncytial Virus infection (RSV), CD38 controls RSV-induced type I/III IFNs. This was demonstrated by the inhibition of CD38’s enzymatic activity that resulted in decreases of the expression of IFN-β, IFN-λ1 and ISG15 in human DCs infected *in vitro* with RSV (62). However, the contribution of CD38 towards cytokine release is still controversial since opposite behaviors have been reported in mouse cells. For instance, CD38 deficient mice had increased levels of TLR4 expression in kidney and enhanced secretion of IL-1β, IL-6, IFN-*γ*, and TNF-α during kidney injury induced by LPS challenge (90). Moreover, CD38 deficiency in unstimulated Raw264.7 mouse macrophages suppressed expression of TLR2 through deacetylation of NF-κB by Sirtuin (SIRT) 1 in an NAD-dependent manner. This phenomenon was also accompanied by increased secretion of inflammatory mediators, including IL-6, IL-1α, CCL2, CCL5, and G-CSF (91). These contradictory results could be explained by differences in stimuli, environment or the type of receptor or enzymatic functions played by CD38 in various circumstances. However, intrinsic differences between CD38’s role in human and mice should be also considered.

Overall, these data point to an active role for CD38 in regulation of cytokine production and indicate that the specific function of CD38 in these processes must be clarified in the future.

### CD38 as a Regulator of NAD^+^ During Inflammation

Nicotinamide adenine dinucleotide (NAD^+^) is a pyridine nucleotide that allows the transfer of electrons during enzymatic reactions (92). NAD^+^ is essential for both glycolysis and oxidative phosphorylation by accepting high-energy electrons from glycolysis and by feeding electrons to oxidative phosphorylation metabolism (93). Fluctuations in NAD^+^ levels compromise homeostasis and impact cellular processes such as transcription, signaling, and cell survival (92). NAD^+^ is as co-substrate needed for the activity of signaling enzymes such as poly (ADP-ribose) polymerases (PARPs), sirtuin deacetylases (SIRTs), and CD38. However, CD38 is the main consuming enzyme of NAD^+^ in mammalian tissues. Supporting this, mice deficient of CD38 have increased NAD^+^ in brain, liver, and muscle tissues, suggesting that CD38 plays a critical role in maintaining NAD^+^ homeostasis (94, 95).

Low levels of NAD^+^ have also been reported during the chronic inflammation associated with aging (96, 97). Aging-associated NAD^+^ changes induce cellular dysfunction and correlate with increased expression and activity of CD38 (97, 98). Thus, it has been suggested that NAD^+^ has a protective role during inflammation. For example, it was shown that NAD^+^ blocks the development of experimental autoimmune encephalomyelitis (EAE) by protecting from axonal degeneration. At the mechanistic level, it was demonstrated that NAD^+^ regulates the differentiation of CD4^+^ T cells and induces the production of the anti-inflammatory cytokine IL-10 by Th1 cells (99). Interestingly, CD38 deficient mice also develop less EAE severity compared with WT mice an effect partially induced by a defect in T cell priming. Furthermore, NAD^+^ precursors (NMN, NAM, and NR) have been linked to anti-inflammatory effects in mouse models of ataxia telangiectasia, another disease characterized by progressive neurodegeneration (100).

Macrophages conduct inflammation through phagocytosis, antigen presentation, and the release of large amounts of pro-inflammatory cytokines and chemokines (101). How NAD^+^ controls these cellular responses in macrophages is another question that has been partially addressed. For instance, Misawa et al. demonstrated that low concentrations of intracellular NAD^+^ promote NLRP3 inflammasome activation and IL-1β release in murine macrophages (102). Similarly, low levels of NAD^+^, in human monocyte-derived macrophages (MDM), induced metabolic changes suppressing oxidative phosphorylation and increasing glycolysis. Furthermore, the reduction of NAD^+^ was companied by a defect in phagocytosis and by increased surface expression of the inflammatory markers CD86 and CD64 while the anti-inflammatory markers CD206 and CD23 were decreased (93). Although these reports did not demonstrate the contribution of CD38 to control NAD^+^ concentration, our laboratory has shown that CD38 is highly expressed in inflammatory MDM and that CD38 controls the release of IL-6 and IL-12. Also, inhibition of CD38 led to a decrease in lactate production, indicating a glycolysis defect (5). Overall, current evidence supports a link between CD38 and NAD^+^ and the idea that CD38-mediated NAD^+^ depletion contributes to inflammation.

It is also important to note that NAD^+^ consumption by CD38 also leads to synthesis of cADPR which can act as an inflammatory molecule by promoting calcium signaling and the activation of several signaling pathways (24). CD38 expression and subsequent cADPR synthesis induced upregulation of COX-2 and prostaglandin E2, a potent chemoattractant (54), in human mesenchymal stem cells (103). Similarly, cADPR controlled murine neutrophils migration toward sites of inflammation, a process dependent on CD38 expression (70, 104). The role of cADPR promoting inflammation is also supported by evidence showing that 8-Br-cADPR, an antagonist of cADPR, suppressed CCL2, reactive oxygen species (ROS), and apoptosis in human retinal pigment epithelium (24, 105).

## CD38 in Autoimmunity

During the last 40 years, CD38 has been shown to regulate multiple components of the inflammatory process, at the level of cell migration, activation, antigen presentation, and cytokine release. Thus, it is not surprising that links between CD38 and chronic inflammation or autoimmunity may exist (106). Autoimmune disorders are a heterogeneous group of diseases that appear as a result of loss of tolerance to self-antigens (107). Autoimmunity is often characterized by lymphocytic infiltration in target tissue and/or circulating autoantibodies (108). Several reports have demonstrated that the absence or expression of CD38 modulates the development of autoimmunity (**Table 1**) (10, 33, 113, 120). The most important findings and controversies on our current understanding on the role of CD38 in autoimmunity are summarized below.

**Table 1:** Role of CD38 during autoimmunity.

Disease	Cells	Possible role of CD38	Refs
Inflammatory Bowel Disease	CD4^+^ Effector T cells CD8αβ^+^ T cells	Unknown	(33, 34, 109, 110),
	Neutrophils Macrophages	Cell recruitment	
Multiple Sclerosis	T cells	Suppresses CD4^+^ Effector T-cell proliferation, T-cell priming	(111, 112)
	Glial Cells (Astrocytes & Microglia)	Promotes glial activation, axonal damage and demyelination, likely probably by regulation of NAD^+^ levels	
	B cells	Regulate release of α-MOG IgG autoantibodies	
Systemic Lupus Erythematosus	B cells	Modulate IL-10 production, α-ssDNA antibodies, and α-nuclear RNP antibodies	(10, 113, 114, 115),
	Myeloid cells	Decrease apoptosis-mediated cell death of Ly6C^lo^ monocytes/macrophages and neutrophils, regulate DCs differentiation	
	Plasma cells	Probable production of autoantibodies	
	T cells	Cell differentiation, decrease cytotoxicity via inhibition of SIRT1/EZH2	
Rheumatoid Arthritis	NK cells	Release of IFN-γ and TNF-α, IL-6 by cyclase activity	(116–119),
	Fibroblasts	Induce proliferation by cyclase activity	
	T regulatory cells	Unknown	
	Plasma Cells	Probable production of autoantibodies	

### Rheumatoid Arthritis

Rheumatoid arthritis (RA) is a systemic autoimmune disorder characterized by autoantibodies, joint inflammation and destruction (121). Autoantibodies are antibodies that react against self-antigens found in cells and tissues. These autoantibodies are produced by plasma cells and are highly specific for target organs in autoimmune diseases (122). In RA, autoantibodies contribute to the pathogenesis by binding to tissue antigens or forming immune complexes that deposit within tissues (123). Therefore, antibody-producing plasma cells and their B cell precursors are thought to play important pathogenesis roles in RA. The pivotal role of B cells in RA has been demonstrated by the fact that rituximab (anti-CD20) proved to be an effective therapy for RA (124). During early rheumatoid and chronic septic arthritis, increases in infiltrating CD38^+^ plasma cells were observed in the synovium of patients, suggesting a role for CD38 in RA pathogenesis (116, 117, 125, 126). Indeed, treatment with plasma cell/plasmablast-depleting anti-CD38 monoclonal antibodies Daratumumab or TAK-079 decreased RA symptoms and disease progression in humans and primates respectively (118, 120). Therefore, CD38 targeting has been proposed as a therapy for RA (120).

A role for CD38 in RA pathology is also suggested by increased CD38 expression in RA patients synovial tissues, high percentages of circulating CD38^+^CD3^+^ and CD38^+^CD56^+^ cells, and high levels of rheumatic factor (120, 127, 128). Furthermore, the expression of CD38 in NK cells has been shown to contribute to RA development through the release of the pro-inflammatory cytokines TNF-α and IFN-*γ* (119). Using a rat model of RA, Wang et al. demonstrated that cyanidin-3-O-glucoside (C3G), a competitive inhibitor of CD38 cyclase activity, decreases the percentage of CD38^+^ NK cells, IL-6 and IFN-*γ* levels in rat peripheral blood and rat synovial fluid. Furthermore, C3G also decreases RA synovial fibroblast proliferation and increases the T regulatory cells proportion in these compartments (119). Similarly, treatment with TAK-079 prevented arthritis development accompanied by a decrease of NK cells, T cells and B cells in blood of cynomolgus monkeys (118) These data support a pathogenic role for CD38 in RA *via* humoral and cellular responses. However, other findings suggest instead that CD38 can also play regulatory roles in RA. For instance, reduced CD38^hi^PDL1^+^CD24^hi^ circulating regulatory transitional B cells have been reported in RA patients (129). In summary, many CD38^+^ pathogenic populations appear to increase during RA. However, the exact impact and mechanism of CD38 during autoreactive immune responses in RA remains obscure.

### Inflammatory Bowel Disease

Inflammatory bowel disease (IBD) is a chronic disorder of the gastrointestinal mucosa encompassing different pathologies, such as Crohn’s disease (CD) or ulcerative colitis (UC) (130). IBD development is thought to be due to unbalanced interactions between genes, environment, immune system and microbiota that result in chronic gut inflammation (131–133). Inflammatory Th1 and Th17 effector T cells are observed infiltrating the gut of IBD patients and IBD animal models (134, 135), suggesting that dysregulated T cell responses may be a pathogenic mechanism (136). In addition, circulating T regulatory cells are reduced in IBD (136).

Several lines of evidence support the involvement of CD38 in the pathogenesis of IBD. Firstly, CD38 is highly expressed in resident and infiltrating immune cells in the colonic mucosa of human and mouse (33, 34). Also, the presence of CD38 has been detected in human T lymphocytes that reside within the lamina propria in the gut (137). Furthermore, the expression of CD38 on intestinal antigen-specific peripheral blood CD4^+^ effector cells has been reported, suggesting a pathogenic role of the molecule (109). In that context, the proportion of CD38^+^ CD4^+^ T cells in humans positively correlate with the mucosal damage marker serum LPS-binding protein (110). These findings suggest that phenotypic changes in circulating CD38^+^ effector T cells are linked to the severity of the disease or its course (138). Further, Schneider et al. showed that CD38 deletion decreases immune cell infiltration and ameliorates DSS-induced colitis (39). However, as has been reported for other autoimmune diseases, CD38 may also actively suppress other pathogenic processes during IBD. In support of this idea, CD38 expression was observed in a population of T cells with regulatory properties in peripheral blood of active IBD patients (138).

At the mechanistic level, an interesting question is how CD38 contributes to IBD development. One possibility is through metabolic regulation of NAD^+^ since increased levels of NAD^+^ have been reported to modulate inflammation in the gut (139). In this sense, a recent proteomic analysis of intestinal tissue from healthy controls and patients with IBD, revealed upregulation of proteins related to NAD^+^ metabolism, including CD38, suggesting that CD38’s NADase activity contributes to modulate NAD^+^ levels during IBD. In this study, they also observed enrichment of CD38 protein expression in inflamed regions of colon mucosa from patients with UC as well as a colocalization of this protein with the marker of macrophages F4/80 (34). Although these findings point CD38 as a regulator of NAD^+^ during IBD, it is not clear how this may impact the development of disease or whether its activity as ADPR cyclase or cADPR hydrolase are also involved. Similarly, the receptor activity of CD38 may also modulate cell responses to induce inflammation however the vast absence of evidence doesn’t allow us to analyze this fact and indicates that more research is required to identify the mechanism driving inflammation by CD38 in the gut.

### Systemic Lupus Erythematosus

Systemic lupus erythematosus (SLE) is an autoimmune disorder characterized by a relapsing and remitting clinical course (140) and autoantibodies against nuclear and cytoplasmic antigens (141, 142). Immunecomplex deposition in multiple organs such as the kidney and skin results in inflammation and tissue damage (143). An increase of CD38^+^ T cells, B cells and monocytes in the circulation of patients with SLE is observed early in SLE (114, 144–146). However, it has been difficult to assign a positive or negative role for CD38 in SLE development, since data supporting both pathogenic and regulatory functions have emerged.

Supporting a pathogenic role, CD38 expression in non-classical monocytes was recently linked to severe active SLE disease in a small group of patients (5), suggesting a potential role as a biomarker and/or a pathogenic role in the development of SLE. Some evidence of a pathogenic role has also been obtained from animal models. For example, CD38 deficiency suppressed SLE in the pristane-induced, type I IFN-dependent, murine lupus model. Clinical benefits were associated with reduction of anti-single-stranded DNA, anti-nuclear ribonucleoprotein (RNP) antibodies, glomerulonephritis, and type I IFN-stimulated gene expression. In this model, CD38 loss also decreased recruitment of-Ly6C^lo^/monocytes/macrophages and Ly6G^+^ neutrophils in the peritoneal cavity (10). This leads to the question of how CD38 may mediate these effects? One possibility is that these effects are mediated by activation of transient receptor potential melastatin (TRPM) 2. ADPR synthetized by CD38 can bind to and active the calcium-permeable channel TRPM2. After activation, the Ca^2+^ flux through TRPM2 induces signaling of cell death pathways that could lead to pristane-induced apoptosis of peritoneal cells, the primary source of autoantigens in this model (10). Another possibility is that CD38 deficiency improved lupus disease through an increase of IL-10-producing splenic B cells and reduction of plasmacytoid dendritic cells and IFN-α production in the peritoneal cavity (147).

While several lines of evidence suggest CD38 may promote SLE disease, evidence to the contrary has also emerged. For instance, CD38 deficiency in the Fas^lpr^/Fas^lpr^ mouse model accelerated disease development while increasing kidney damage and circulating IgG. While the mechanism for disease protection is unclear, altered CD4^+^CD25^+^ and CD4^+^ single-positive thymocyte and CD8^-^ dendritic cell proportions were observed in CD38 deficient mice. These observations suggest that CD38 modulation of T-cell and dendritic cell differentiation may suppress lupus autoimmunity (113). Another line of evidence suggesting regulatory roles of CD38 in B cells again originates from the lpr SLE model. High CD38 expression is observed in CD1d^hi^ CD5^+^ regulatory B cells in this model. Finally, treatment with an agonistic anti-CD38 stimulus during LPS activation increased the percentage of regulatory B cells and, of relevance to regulatory function, their ability to produce IL-10 (115).

In summary, controversial data supporting both beneficial or pathogenic roles for CD38 in SLE disease and or animal models have arisen. These reports, however, clearly indicate that CD38 plays important roles in SLE and indicates the need for a better understanding of how CD38 contributes to immune cell physiology and SLE pathobiology.

### Multiple Sclerosis

Multiple sclerosis (MS) is a chronic autoimmune disease of the central nervous system (CNS) that leads to multiple neurological signs, from sensory/cognitive deficits to motor disability (111, 148). MS is characterized by axonal demyelination as a consequence of the infiltration of circulating inflammatory cells into the CNS, including T cells, B cells, and myeloid cells (149, 150). Together, these cells induce tissue damage through secretion of soluble mediators and oxidative stressors (151, 152). The first evidence that CD38 may be involved in this disease resulted from efforts to identify pathogenic factors in the experimental autoimmune encephalomyelitis (EAE) model. CD38 was specifically induced during active myelin oligodendrocyte glycoprotein (MOG) in a rat model of EAE disease (111). To demonstrate the contribution of CD38 to pathogenesis, EAE disease was evaluated in CD38 deficient mice. CD38 deficiency reduced EAE severity, and this effect appears to be mediated by T and B cells (111). Supporting this idea, MOG-specific T cell responses were reduced in absence of CD38, indicating T-cell priming/proliferation defects. Humoral responses are also affected, as evidenced by decreased anti-MOG IgG autoantibody release in CD38 deficient mice (111).

The molecular mechanisms behind disease modulation are unclear, albeit NAD^+^ consumption by CD38 may be a contributing factor. During chronic CNS inflammation in mice, NAD^+^ levels are altered (153–155), promoting neuron demyelination (99, 153). Furthermore, it has been shown that the absence of CD38 significant increase NAD levels in mice brain (156, 157). Thus, as one of the main regulators of tissue NAD content, CD38 could have a profound impact on neuronal damage. Recently, the bis-cyclohexanone oxaldihydrazone (CPZ)-induced demyelination model revealed that CD38 deficiency suppresses glial activation, axonal damage, and demyelination. This effect was hypothesized to be mediated *via* enhanced levels of NAD^+^ (112). Under homeostatic conditions, astrocytic CD38 regulates murine astrocytes maturation and oligodendrocytes (OL) differentiation (158). However, under CPZ-induced demyelination, absence of CD38 impaired OL repopulation (112).

The expression of CD38 in other cells that contribute to MS pathogenesis has also been demonstrated (156). For instance CD38’s expression in human astrocytes positively regulates IL-6 and CCL2 production while CD38 deficiency in rat astrocytes increases H_2_O_2_-induced cell death (156, 159, 160). Similarly, activated microglial cells also express CD38. Here, CD38 positively regulates release of the proinflammatory cytokines IL-1β, TNF-α and IL-6 as it was demonstrated in a murine model of LPS-activated microglia (161).

Overall, these studies support a role for CD38 in MS physiopathology. However, more studies are needed to understand the cellular and molecular mechanisms behind CD38-mediated disease pathology.

## Tools for CD38 Study: Successes and Remaining Challenges

**Figure 3:**
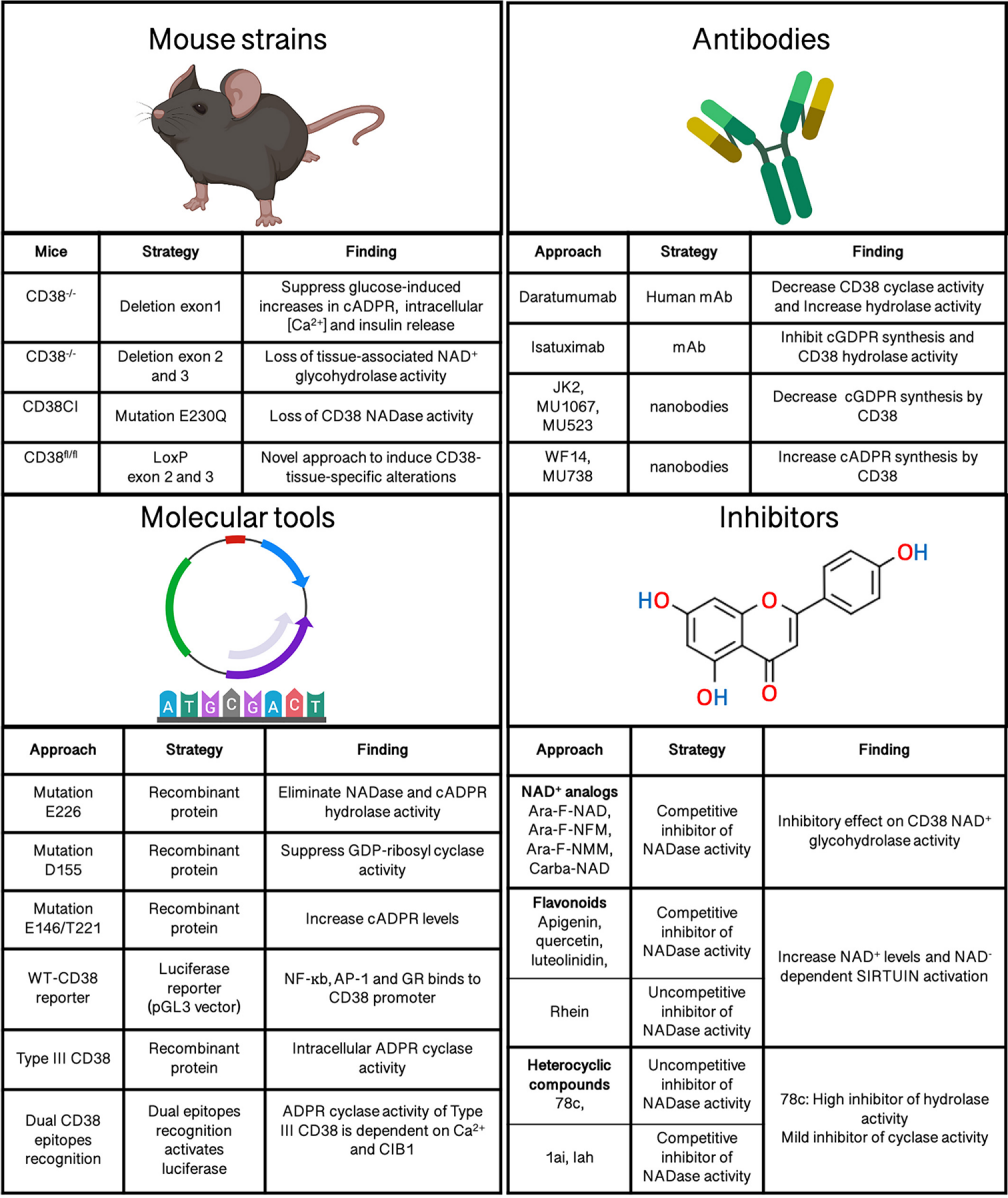
Molecular tools to analyze CD38’s functions.

Better understanding of the specific effects and mechanisms by which CD38 modulates inflammation and autoimmune disease will require specialized tools and resources. Fortunately, several tools have already been generated (**Figure 3**), with the potential to provide novel insights into CD38’s biology and mechanism in physiology and disease. These tools include recombinant proteins, monoclonal antibodies, inhibitors, transgenic and knockout mouse strains (12, 162–167). Currently available tools and models, as well as remaining challenges, are discussed below

### Recombinant Proteins

A number of CD38 molecular tools have been instrumental in our understanding of how CD38 expression is controlled or specific aspects of CD38’s enzymatic function/s. These tools include reporter systems carrying CD38 promoter sequences and recombinant wild-type or mutated CD38 proteins/peptides fragments (12, 168, 169).

Mutated CD38 proteins and fragments have provided insights into the CD38 domains mediating crucial enzymatic and CD31 binding activities. For example, in an effort to modulate CD38 enzymatic activity, site-directed mutagenesis studies of human CD38 revealed E226 as the essential residue for CD38’s catalytic activity. Substitution of E226 with D, N, Q, L, or G completely eliminates NADase and cADPR hydrolase activity. Instead, residue D155 was found to be crucial for cyclic ADP-ribosyl synthesis (12, 170). On the other hand, early evidence documented the intracellular localization of CD38. To determine whether intracellular CD38 has enzymatic activity, a soluble form of human CD38 that cannot localize to the membrane was engineered. Interestingly, this protein synthesized intracellular cADPR using cytosolic NAD as substrate. Also, mutations at E226, E146 and T221 in soluble CD38 confirmed that E226 is essential for cADPR production and revealed that E146A and T221F mutations in turn increase cADPR synthesis (168). Together these findings support the idea that intracellular CD38 is functionally active. As mention earlier, CD38 can take up type II or type III orientations on the cell membrane, depending on whether the C-terminal catalytic site of CD38 faces the extracellular or cytosolic compartment cytosol (171). However, the functional significance of type III orientation was not confirmed until recently thanks to a mouse type III CD38 construct. This tool revealed hydrolase/cyclase/enzymatic activity and the requirement of H_2_O_2_/Nox4 for intracellular cADPR generation. Additional type III CD38 mutant constructs revealed that C164 is an essential aa for cADPR synthesis and Ca2^+^ signaling (172). Defining the functional activity of type III CD38 solved the topological paradox of CD38, namely why CD38’s catalytic domain faces extracellularly while its NAD substrate is cytosolic and its cADPR product targets intracellular pathways. These are some examples of how recombinant protein strategies have substantially advanced our understanding of CD38 biology.

Luciferase CD38 reporters have also been useful to dissect the pathways and transcription factors controlling the transcriptional regulation of CD38 (173). The induction of CD38 following cell stimulation was analyzed following this approach using a putative CD38 gene promoter fragment cloned into the pGL3 luciferase vector. This approach identified the TNF-α-dependent binding of NF-κb, AP-1 and glucocorticoid receptor (GR) to the CD38 promoter, linking these transcription factors to CD38 expression (169). Without a doubt, this strategy is a powerful tool since identifying the elements controlling or inducing CD38 expression may shed light into why CD38 is linked to heterogeneous responses under similar immunological contexts.

### CD38 Pharmacologic Inhibitors

Several molecules with CD38 inhibitor activity have been developed (98, 167, 174). These compounds bind to CD38’s active site, modulating its activity, and can be classified in three classes, namely NAD^+^ analogs, flavonoids, and heterocyclic compounds (25).

NAD^+^ analogs are thought to act as CD38 inhibitors *via* substrate competition. Specifically, the mechanism of action of these molecules involves competitive inhibition of NAD glycohydrolase activity (175). NAD^+^ analogs have low affinity to CD38 and can be subclassified into covalent and non-covalent inhibitors. Examples of NAD analogs include Ara-F-NAD, Ara-F-NFM, Ara-F-NMM, Carba-NAD, etc (167, 176). Unfortunately, these NAD^+^ derivatives are charged molecules with limited cell permeability, reducing their usefulness as CD38 modulatory tools (167).

Flavonoids are CD38 inhibitor compounds naturally produced by plants (174, 177). Flavonoid inhibitors include apigenin, quercetin, luteolinidin and 4,5-dihydroxyanthrquinone-2-carboxyl acid (Rhein) (174). Some CD38-flavonoid inhibitors act as competitive agonists with NAD, what results in increased NAD^+^ and NAD-dependent sirtuin activation (178). However, the mechanism of action of most flavonoid inhibitors is yet unknown.

Finally, several heterocyclic compounds act either as competitive or uncompetitive inhibitors of NADase activity *via* non-covalent reversible binding to CD38. These compounds are derived from 4-amino-quinoline and include 78c, 1ai, and Iah (19). Furthermore, they are positioned as better candidates than flavonoids and NAD^+^ analogs due to their specificity, improved pharmacokinetics, oral bioavailability and biological stability. The lead inhibitor 78c has demonstrated potent, specific and uncompetitive CD38 inhibitory activity. 78c preferentially inhibits CD38 NADase activity and is 10-fold less potent against cyclase than hydrolase activity. Interestingly, 78c rescued age-related NAD^+^ levels in tissues improving metabolism and physiological characteristics related to aging as glucose homeostasis, age-related skeletal muscle or cardiac dysfunction (98).

### Regulation of CD38 Enzymatic Activity by Antibodies

CD38 is a diagnostic and prognostic biomarker in hematologic malignancies and multiple myeloma (MM) (179). As a consequence, several anti-CD38 monoclonal antibody (mAbs) therapies have been developed and are at either preclinical or clinical stages of drug development (18, 166, 180). Daratumumab (DARA) was the first fully humanized immunoglobulin G1 kappa mAb targeting CD38 but additional therapeutic antibodies such as isatuximab, MOR202 and TAK-079 have been developed. Although the anti-tumor activity mechanism is still under investigation, there is evidence that anti-CD38 mAbs can kill CD38-overexpressing cancer cells by multiple processes. These processes include complement-dependent cytotoxicity, antibody-dependent cellular cytotoxicity, antibody-dependent phagocytosis, tumor cells apoptosis and regulation of enzymatic activity of CD38 (181).

Besides their therapeutic activity in cancer, anti-CD38 mAbs may also provide interesting experimental tools to dissect CD38’s functions in other contexts. In vitro studies suggest that DARA could modulate CD38’s enzymatic activity (166, 180). In fact, a report indicates that DARA decreases cyclase activity while stimulating hydrolase function (166). However, these observations need further validation. Stronger evidence is available for Isatuximab as an inhibitor of CD38 enzymatic activity (182). In vitro, Isatuximab suppressed the production of cyclic GDPRibose (cGDPR) from NGD^+^ (183). In addition, Isatuximab treatment inhibited the hydrolase activity of CD38 in a dose-dependent manner (183).

Moreover, classical antibodies, nanobodies, and single domain antibodies against CD38 have been generated. These tools have improved CD38 binding and blocking capabilities while providing improved tissue penetration over conventional antibodies. Interestingly, several nanobodies modulate CD38’s catalytic activity. Fumey W et al, identified three nanobodies (JK2, MU1067, and MU523) able to decrease CD38-dependent cGDPR synthesis while two others (WF14 and MU738) conversely enhance CD38-catalyzed synthesis of cADPR (165). Although the evidence is still limited, these findings suggest that nanobodies could be also used to target enzymatic activity of CD38. Nanobodies have also allowed to study type II vs Type III membrane orientation of CD38. Nanobodies recognizing two different epitopes within CD38’s C-terminal domain were fused to distinct luciferase fragments and expressed in the cellular cytosol. When both epitopes are recognized by the nanobodies, the two luciferase fragments are sufficiently close to produce luminescence. This strategy demonstrated that type III CD38 exists as a transmembrane protein with ADPR cyclase activity. In addition, it showed that type II CD38 activation is dependent on the interaction with cytosolic Ca^2+^ and integrin-binding protein 1 (CIB1) (17).

### CD38 Animal Models

In 1998, the groups of Howard and Okamoto developed the first two CD38 deficient (CD38^-/-^) mouse models, allowing to analyze the *in vivo* role of CD38 (162, 163). Both models were generated on a C57Bl/6J genetic background, albeit through different genetic approaches. Okamoto’s model used a homologous recombination strategy that deleted CD38’s exon 1, resulting in loss of CD38 transcript and protein. On the other hand, Howard’s model was designed to delete exons 2 and 3 encoding CD38’s putative active site. Both CD38 deficient mice revealed alterations consistent with the loss of CD38’s enzymatic activity. For instance, Okamoto’s model revealed impaired intracellular calcium and cADPR in pancreatic cells (163) while Howard’s model shown reduced NAD^+^ glycohydrolase activity in liver, spleen, and brain (162).

CD38^-/-^ mice have been instrumental to our understanding of the enzymatic activity of CD38, reveling its multiple roles as NADase, ADPR cyclase, and cADPR hydrolase, as well as its contribution to regulate intracellular calcium mobilization/signaling through the second messenger cADPR (70). Furthermore, additional functions of CD38 in signaling, adhesion and cell migration have been also identified through these models (70, 71). CD38^-/-^ mice have also been excellent tools to demonstrate the crucial role of CD38 in immunity to *L. monocytogenes* (45), *S. pneumoniae* (68)*, M. avium* (184)*, S. Thyphimurium* (89), *and E. histolytica* (66). Although most of these findings have been reported in immune cells the contribution of CD38 to physiology of non-immune cells have been also target of study utilizing these constitutive CD38^-/-^ mice. For instance, CD38 deficient pancreatic beta cells displayed impaired glucose-induced cADPR production and Ca^2+^ signaling, resulting in impaired insulin secretion. Similarly, CD38 was also reported to mediate Ca^2+^ signaling and activation of hepatic stellate cells that contribute to liver fibrosis (185).

Besides single CD38 knock-out (KO) mice on the B6 background, the CD38 mutation has been combined with other genetic backgrounds or other gene KOs. For a variety of reasons, these models have provided information on CD38’s impact on inflammatory and autoimmune processes. Due to the observed effects of CD38 on pancreatic beta cell biology, the CD38 KO mutation was crossed onto the autoimmune diabetes-prone NOD/Lt background. The resulting NOD-CD38^-/-^ mice suffer from accelerated type 1 diabetes development as consequence of NAD-induced apoptosis in T cells (164). ADP-ribosyltransferase 2 (ART2) is an ectoenzyme that catalyzes the transfer of ADP-ribose group from NAD^+^ to target proteins such as P2X7, a purinoreceptor that elicits T cell apoptosis (186). Hence, the double deficient NOD-CD38^-/-^ART2^-/-^ mice were generated demonstrating that accelerated diabetes observed in NOD-CD38^-/-^ mice was dependent of ART2 activity to mediate T cell apoptosis through P2X7 (164). Similarly, ART2^-/-^CD38^-/-^ and TRPM2^-/-^CD38^-/-^ double KO mice were used to study the contribution of CD38 to pristane-induced lupus. This approach demonstrated that Ly6C^hi^ monocyte recruitment to inflamed tissues requires CD38 expression. In addition, these experiments provided evidence that CD38 promotes apoptosis of monocyte/macrophages in a TRMP2-dependent manner during experimental lupus (10).

Double-KO strategies have also shed light on the contribution of CD38 to aging-associated inflammation and immune responses against pathogens. During aging, low-grade inflammation, known as inflammaging, and a decline in NAD^+^ levels is observed (23). Interestingly, NAD levels are restored in absence of CD38 and this induces an increase of SIRT3 activity, a mitochondrial enzyme that uses NAD^+^ as a substrate. In order to determine to what extent CD38 contributes to inflammaging, a CD38^-/-^SIRT3^-/-^ double KO mice model was developed. This model revealed that CD38’s NADase activity induces an age-related NAD decline that drives mitochondrial dysfunction through SIRT3, as SIRT3 deletion in CD38^-/-^ mice reversed the effects observed in this model (97). Similarly, the contribution of CD38 to immune responses against pathogens was analyzed by Partida-Sanchez et al. taking advantage of crossed CD38^-/-^ mice onto the lymphocyte-deficient Rag2^-/-^ background. This strategy demonstrated that myeloid cells expressing CD38 are responsible for controlling *S. pneumoniae* infection and dissemination (70).

Constitutive CD38^-/-^ mouse models have provided really valuable information on CD38 biology. However, given the ubiquitous expression and multifunctional nature of CD38 molecule, these models have the disadvantage of impacting many tissues and CD38 functions at the same time. Thereby, the ability to generate tissue specific CD38-deficient models or suppress specifically one of CD38 function is essential to understanding CD38 physiology *in vivo*. With this in mind, mice with LoxP flanking of exon 2 and 3 of the CD38 gene were recently generated to induce specific deletions of CD38 in airway smooth muscle cells (187). This approach helped describe how CD38 regulates hypoxia-induced apoptosis by SIRT1 and the p53 signaling pathway (187). At the same time, this conditional KO model opens many opportunities to analyze CD38’s tissue-specific roles. Regarding functional activity of CD38, Tarragó et al. developed a promising Knock-in mouse to elucidate the role of CD38 in age-related metabolic dysfunction mediated by NAD^+^ decline. This model eliminated CD38 catalytic activity by the punctual mutation E230Q in the CD38 gene. Catalytically inactive CD38 (CD38-CI) mice preserved CD38 protein expression but had null CD38 NADase activity thus ensuring specific analysis of CD38 at the enzymatic level (98).

Overall, the animal models discussed in this section provide evidence of the ever-expanding array of biological tools available to analyze the function of CD38 *in vivo* and its impact in autoimmune and immune mediated diseases.

## Concluding Remarks

Constant efforts to understand CD38’s biology have allowed us to improve our knowledge of this complex molecule. CD38 was originally identified as a protein expressed on the surface of T cells but nowadays, we know that CD38’s function goes beyond a single cell or serving as a simple molecular marker. Indeed, CD38 plays critical roles in human physiology by modulating homeostasis, inflammation, or autoimmune responses in our body. Hence, CD38 has been proposed as a prognostic marker in some pathologies and several therapeutic anti-CD38 monoclonal antibodies are currently being developed for the treatment of malignancies such as melanoma. However, the ubiquitous localization of CD38, its multiple functions, and its dual membrane orientation represent challenges for mechanistically understanding the contribution of CD38 to health and disease. As a consequence, several outstanding questions remain. For example, it is unknown how CD38 is able to mediate regulatory and pathogenic responses in the same disease. Similarly, it is not completely understood which cellular events are triggered by each of CD38’s functions as well as what are their biological consequences. Some molecular tools have been developed that could help to partially answer these questions; however, more novel strategies are needed to be able to analyze and modulate each of CD38’s functions independently. Moreover, there are several immune cells, tissues and diseases where the role of CD38 has not been characterized. In summary, we have witnessed great progress in our knowledge of CD38 biology, and there is still much more to learn about this fascinating protein.

## Author Contributions

ZP-Q and MG-d-A conceptualized the manuscript, reviewed literature, and wrote the review. PN contributed ideas and wrote the review. ZW reviewed the literature. ZP-Q and ZW designed figures and table. All authors contributed to the article and approved the submitted version.

## Funding

MG-d-A is supported by funds from the NIH National Institute of Allergy and Infectious Diseases grants 1R21AI127354 by R21AI127354; 1R03AI151769 by R03AI151769; American Association of Immunologists, Careers in immunology by Association of Immunologists-Careers in immunology; subaward GRT00050231 by R01AI137525.

## Conflict of Interest

The authors declare that the research was conducted in the absence of any commercial or financial relationships that could be construed as a potential conflict of interest.
